# Supplementary material for: Patterns of Intron Gain and Loss in Fungi
Source: PLoS Biol. 2004 Nov 30;2(12):e422. doi: 10.1371/journal.pbio.0020422 (PMC532390; doi:10.1371/journal.pbio.0020422)
Supplement: Table S1 — Also available at http://genes.mit.edu/NielsenEtAl/. (4.3 MB ZIP). [file pbio.0020422.st001.zip › NielsenEtAl/html/619.html]

AN2885.1.NCU09995.1.MG04489.1.FG00385.1


```
 CLUSTAL W (1.82) Multiple Sequence Alignments - Introns Inserted


Sequence 1: NCU09995.1	103 aa
Sequence 2: FG00385.1	101 aa
Sequence 3: MG04489.1	101 aa
Sequence 4: AN2885.1	106 aa
Alignment Length: 106 aa
Number Identitical Residues: 68 aa
Alignment Score (without introns) 2839


MG04489.1 	MPKAAT--KKSAKPEK-RRAKKD~PMAPKRGLSAYMFFANEQRDNVREENPGVTFG~QVG
NCU09995.1	MPKAAAKSKTTGKVEK-RRAKKD1PNAPKRGLSAYMFFANEQRENVREENPGVSFG~QVG
FG00385.1 	MPKAAAPAKRATRT---KRAKKD1PNAPKRGLSAYMFFANEQRENVREENPGISFG~QVG
AN2885.1  	MPKANPTRKTKATRETGGRKKKD1PNAPKRGLSAYMFFANDNRDKVREENPGISFG1QVG
          	**** .  *     ... * *** * **************::*::*******::** ***

MG04489.1 	KILGERWKALSDKQRAPYDAKAAADKKRYEDEKAAYQ0AGGDED--ESS
NCU09995.1	KILGERWKALSDKQRAPYEAKAAADKKRYEDEKQAYN0AEADEE--ESS
FG00385.1 	KLLGERWKALNEKQRAPYEAKAAADKKRYEDEKQAYN0ADQEEE--ESS
AN2885.1  	KMLGEKWKSLSDKERKPYEDKAAADKKRYEDEKAAYK0AGEAEEDEESS
          	*:***:**:*.:*:* **: ************* **: *   *:..***
```
